# Underfed Children

**Published:** 1906-07-14

**Authors:** 


### Underfed Children.

The report of the Joint Committee on Underfed School Children, constituted by the London County Council, is a very interesting and important document. It would appear that what may be called the "free meals" movement was mainly set on foot by persons who may perhaps be best described as "acting foolishly, but meaning well," and who are the natural victims of certain predatory classes of the community. In reply to an inquiry from the Fulham Guardians, lists of names of children in want of food were supplied from seven schools, and similar lists from 11 schools were supplied to the Wandsworth Guardians. Official inquiry was then made into the facts; and, in the great majority of cases, the reports of the relieving officers differed widely from the information collected by the relief sub-committees. Out of 89 cases investigated by the Fulham Guardians 76 were reported, in the opinion of the relieving officer, not to be underfed; and a similar report was made with regard to 60 out of 88 cases in Wandsworth. The general conclusion of the Committee is that the funds contributed by the benevolent public will be adequate to meet the needs of the case; and if the Provision of Meals Bill, when passed into law, throws on the local education authority the responsibility of providing food for necessitous children (but not for all children) at the cost, not of the rates, but of voluntary subscriptions, the Committee believe that much benefit will come to the children, and that the system may be so worked as not to cause any weakening of the sense of parental responsibility.

With the main conclusions of this report, which seem, on the whole, to rest on grounds of common sense and probability, it would be difficult not to agree. The chief fact which it brings into prominence is that, after 35 years of compulsory "education," education received by many of the grandparents and by all the parents of the children now attending elementary schools, the great majority of the mothers of the industrial classes are no better qualified for the fulfilment of their domestic duties than were women of the same position a hundred years ago. They have largely inherited the slender sense of responsibility, with regard to the claims of parentage, which was the natural outcome of the old Poor-law system, the system under which the early marriage of labourers was promoted, and each additional child was welcomed as a source of income from the parish. In opposition to this view, there is unquestionably a growing reaction, and an increasing sense of the fact that children are hostages to the State, and the basis of the future wealth and prosperity of the nation; and it is this feeling, if England is to continue a great country, that the educators of the working population should strive sedulously to cultivate. The industrial classes are surrounded by many enemies, whose constant endeavour it is to exploit them for the attainment of private ends. They are grossly flattered by politicians, who at the same time practise upon their want of information and seek to rise to power upon their shoulders. They are grossly flattered by preachers, declaiming in the interests of their respective sects, and constantly dwelling upon the importance of verbal tenets, to the neglect of decent and well-ordered lives. Their very education, such as it is, delivers them over to every quack, religious, political, or other, who can find an opening for his views in a halfpenny newspaper, or to every lying advertiser who strives to swindle them out of their money by his descriptions of his worthless wares.

The improvement of home conditions would, as the report we are considering declares, be the most important step that could be made towards the ultimate attainment of better comprehension of the problems of life; but the first step in this direction should be in such a modification of the existing school system as to make it afford instruction in matters which the children of the wealthier classes learn, as a rule, in the nursery, but which the children of the poor may never learn at all. They have no knowledge of the importance of cleanliness or fresh air, or what may be called the principles of conduct, and the inevitable character of the consequences which misconduct involves. The want of this knowledge alone is perhaps the most potent of all the influences which add to the ranks of the unemployed, or which convert the unemployed into the unemployable. Except in cases manifestly calling for assistance, parents should be compelled to maintain their children, and should be punished for any obvious dereliction of duty with regard to them.

